# Bacterial Survival Amidst an Immune Onslaught: The Contribution of the *Staphylococcus aureus* Leukotoxins

**DOI:** 10.1371/journal.ppat.1003143

**Published:** 2013-02-21

**Authors:** Francis Alonzo, Victor J. Torres

**Affiliations:** Department of Microbiology, New York University School of Medicine, New York, New York, United States of America; University of North Carolina at Chapel Hill School of Medicine, United States of America

The success of *Staphylococcus aureus* as a human pathogen is influenced by its ability to elaborate factors that prevent infection resolution by the host immune system. Such immune-altering factors include complement inhibitory molecules, antibody binding proteins, super-antigens, as well as potent cytolytic peptides and pore-forming toxins. Here, we discuss one class of immune cell-targeting toxins, the bi-component leukotoxins. These toxins are believed to form octameric oligomers of alternating subunits on the surface of host cells and insert β-barrel pores into cell membranes leading to osmotic imbalance and cell lysis [1]. We will discuss the reemerging interest in leukotoxins as potent virulence factors with defined cellular targets, the implications of their lethal and sublethal cellular effects, as well as challenges that have restricted understanding of their functional activity in vivo, while emphasizing areas of interest for future exploration. In addition, we highlight studies supporting the development of antileukotoxin antibodies and immunization strategies as potential modalities to counter *S. aureus* infection.

## PVL and Beyond: A Reemerging Interest in Immune Cell-Targeting Toxins

**Figure 1:**
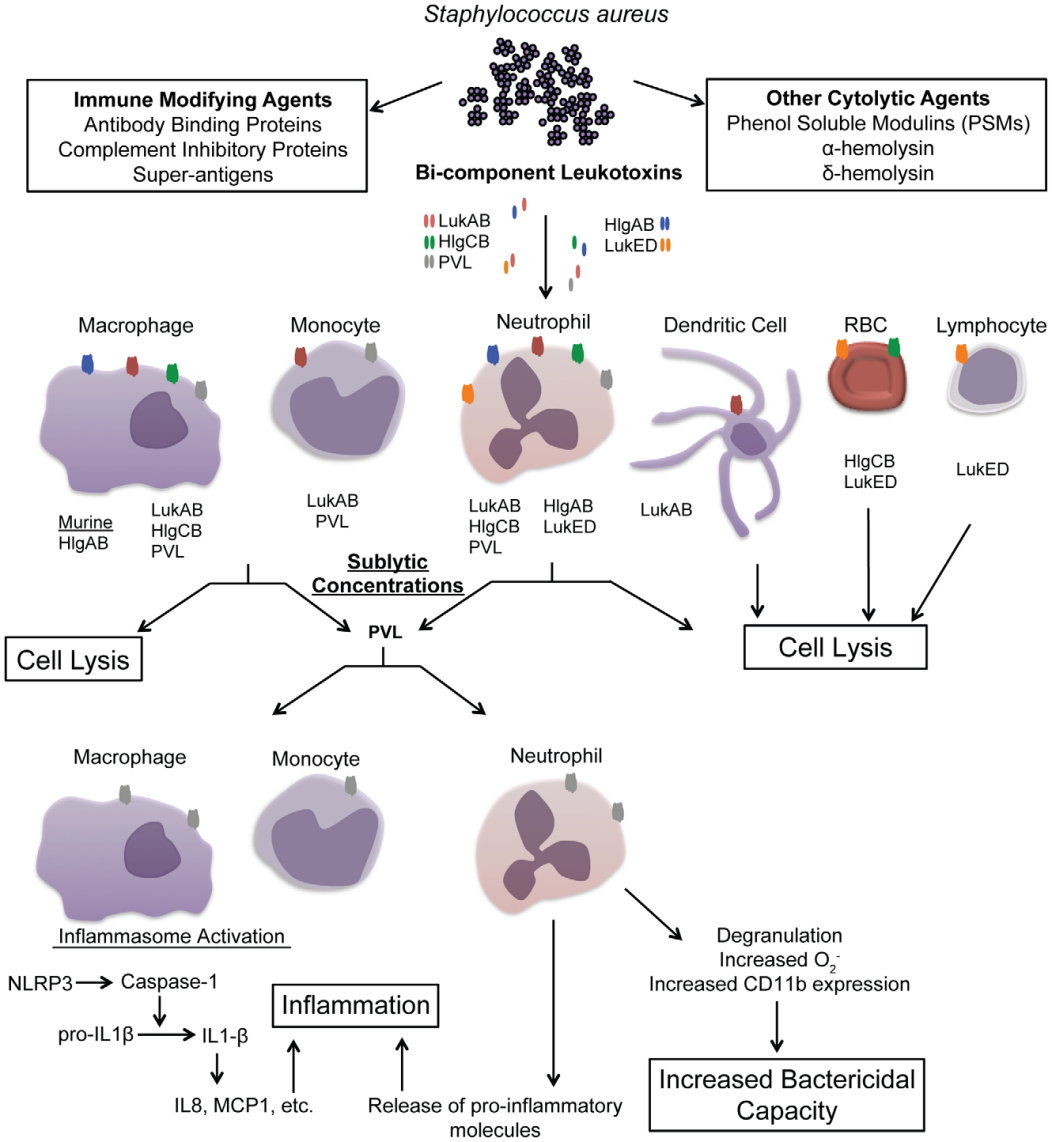
Overview of *S. aureus* leukotoxin action on host immune cells. The above schematic illustrates our current understanding of leukotoxin targeting and the functional consequences of toxin activity on specific immune cell types. Upon encountering host immune cells, *S. aureus* elaborates numerous molecules that facilitate escape from bacterial killing. The bi-component leukotoxins (LukAB, red; HlgCB, green; PVL, gray; HlgAB, purple; LukED, orange) are one class of lytic molecules that directly target and kill immune cells. The cells types currently known to be targets of each leukotoxin are shown. While the leukotoxins possess potent lytic capacity on their cellular targets, evidence also suggests a sublytic influence of PVL. The downstream effects of sublytic toxin activity, including inflammasome activation and enhanced bactericidal activity are shown.

The most studied of the leukotoxins produced by *S. aureus* is the Panton-Valentine Leukocidin (LukSF/PVL). Interest in this toxin stems from its prevalence among current epidemic strains of community-acquired methicillin-resistant *S. aureus* (CA-MRSA) [2]. Indeed, epidemiological evidence exists to link PVL to a number of diseases including skin and soft tissue infections as well as necrotizing pneumonia, for which CA-MRSA is so notorious [2]–[6]. Experimentally, assessment of the contribution of PVL to pathogenesis has been plagued by conflicting results, owing to the apparent species specificity of toxin action [7]. Despite these experimental difficulties, PVL has been linked to both necrotizing pneumonia and soft tissue infections using rabbit infection models, although its actual contribution to skin and soft tissue infection remains controversial [8]–[10]. However, PVL is only one of a family of four additional leukotoxins present in strains causing human disease. These include the gamma hemolysins (HlgAB and HlgCB), leukocidin ED (LukED), and leukocidin AB (LukAB, otherwise known as LukHG [11], [12]) (Figure 1). A resurgence of interest in these leukotoxins, which are conserved in a greater percentage of clinical isolates, has led to the discovery of potential distinct roles for each toxin in *S. aureus* pathogenesis.

Though identified ∼10 years ago, LukED had only been evaluated in terms of its in vitro capacity to lyse human and rabbit neutrophils as well as red blood cells (Figure 1) [13], [14]. Recently however, LukED has been found to contribute to *S. aureus* pathogenesis upon murine systemic infection due, in part, to toxin killing of phagocytic leukocytes in vivo [15]. The newly identified leukotoxin LukAB was also shown to contribute to distal tissue colonization upon infection with sublethal doses of MRSA [12]. Additionally, among earlier reports of a role for Hlg in septic arthritis and endophthalmitis [16], [17], recent evidence from Malachowa and colleagues suggests a role for this toxin in bloodstream infection [18]. Together, these data indicate leukotoxins likely contribute to multiple *S. aureus* disease states in vivo. Future investigation into the contribution of each leukotoxin to *S. aureus* pathogenesis using multiple infection conditions and animal models will serve to delineate each toxin's capacity to promote disease.

## Challenging the Proposition of Strict Functional Redundancy

*S. aureus* leukotoxins exhibit lytic activity on host neutrophils, although some have greater perceived potency than others (Figure 1). Early studies gave considerable attention to this apparent redundancy in toxin targeting using primary human and rabbit neutrophils [19], [20]. However, recent efforts are now moving toward investigation of the full repertoire of cells killed by each leukotoxin, with the premise that each may be unique in both the breadth and specificity of its cellular targets despite significant similarities at the amino acid and structural level. Studies by Holzinger et al. confirmed earlier work describing the lytic capacity of PVL on neutrophils and monocytes, but not lymphocytes [21], [22]. In addition, they demonstrated the lytic capacity of PVL on macrophages (Figure 1) [21]. HlgCB is toxic toward neutrophils and macrophages but also exhibits lytic activity on red blood cells [23]. HlgAB, on the other hand, is nontoxic toward human macrophages but is potent on murine macrophages (Figure 1) [23]. LukAB is toxic toward neutrophils, monocytes, macrophages, and dendritic cells, but not the T cell line Jurkat [12]. LukED is active against human and rabbit neutrophils, rabbit red blood cells, as well as murine leukocytes (Figure 1) [13]–[15]. The subtle differences in leukotoxin activity on specific cell types imply cellular recognition via unique factors. Thus, inferring direct relationships between the potency of one leukotoxin and another is challenging, as abundance or accessibility of specific cellular targets may vary significantly on host cell surfaces. Deciphering the reasons for varied potencies of the leukotoxins on similar cell types as well as their mechanisms of cellular targeting will prove valuable in future attempts to equate leukotoxin function with pathogenic outcomes.

## The Lytic Versus Sublytic Hypothesis

It was recently demonstrated that the killing of host phagocytes during systemic infection of mice with *S. aureus* is dependent on LukED production [15]. Thus, the lytic capacity of LukED is likely a biologically relevant process during *S. aureus* pathogenesis. Other studies with PVL demonstrate that leukotoxins may also elicit cellular effects at sublytic concentrations (Figure 1) [21], [23]–[28]. Notably, PVL induces inflammasome activation of both monocytes and primary macrophages at sublethal doses [23]. Inflammasome activation in this context is believed to contribute to the inflammatory response and subsequent neutrophil recruitment during necrotizing pneumonia (Figure 1) [23]. Low concentrations of PVL also appear to prime PMNs for increased bacterial killing by promoting neutrophil activation (Figure 1) [24]. Unfortunately, investigation of the influence of sublytic toxin concentrations on leukocytes in vivo has only been studied using PVL in murine models [29], [30]. Such studies are limited due to an inability of PVL to lyse murine cells. Thus, while the sublytic effects of PVL on leukocytes are intriguing, the in vivo consequences of such effects in the presence of active toxin are not understood. Lending credence to the hypothesis that *S. aureus* toxins influence cellular signaling during infection, the prototypical pore-forming cytotoxin alpha hemolysin targets macrophages to induce inflammasome activation in vivo [31], [32]. It is possible that within a host both lytic and sublytic concentrations of the bicomponent leukotoxins are also encountered depending on the site and context of infection. Studies using active toxins amenable to small animal models (such as LukED) may prove valuable in determining the consequences of such sublytic effects.

## Overcoming Species Specificity to Investigate Leukotoxin Function in Vivo

As mentioned, studies of PVL function in vivo have been complicated by the species specificity associated with cellular targeting. PVL has negligible lytic activity on murine neutrophils but is potent on human and rabbit cells [33]. Similar studies have demonstrated poor lytic activity of LukAB on murine and rabbit neutrophils, but potent activity on human neutrophils [12], [25]. Interestingly, LukAB still influences the pathogenesis of MRSA in murine systemic infection models [12]. Future work aimed at deciphering the mechanism by which LukAB facilitates pathogenesis in murine models will allow a better assessment of this toxin's activities in vivo. Additionally, HlgCB kills human macrophages but exhibits little lytic activity on murine macrophages [23]. Thus, studies using murine models to evaluate these particular toxins in vivo are best interpreted in light of their nonlytic effects. For this reason, many PVL studies are now conducted using rabbit models of infection [8], [9]. In contrast, LukED is toxic toward murine, rabbit, and human leukocytes and is thus amenable to in vivo studies using murine models of infection [15]. Indeed, the lytic activity of LukED was recapitulated on phagocytic leukocytes in vivo [15]. Thus, future studies of LukED are likely to provide a robust model of leukotoxin function in vivo. The additional finding that HlgAB is lytic on murine macrophages supports assessment of this toxin using murine models [23].

## The Leukotoxins as Valuable Vaccine and Therapeutic Targets

Evidence indicating a role for each of the leukotoxins in the greater virulence of *S. aureus* implies potential value in targeting these molecules to counter infection. Leventie et al. have generated humanized heavy-chain-only antibodies and diabodies against PVL that show promise in their ability to neutralize the damaging effects of the toxin in vivo [34]. These same anti-PVL antibodies also block the activity of HlgCB on host cells [34]. Dual neutralization by these antibodies is perhaps not surprising due to the high degree of sequence similarity among leukotoxins but serves as proof that it is possible to generate single antibodies or a subset of antibodies with the ability to neutralize multiple leukotoxins, thereby blunting disease progression [35]. Vaccination of mice with PVL has likewise demonstrated promise toward reducing the pathogenic outcome of *S. aureus* infection [36], though other studies, which passively immunized mice with serum from PVL-immunized rabbits, lead to increased virulence for some strains [30]. In either case, these murine studies should be interpreted with caution given the low level of PVL lytic activity on murine cells. Even so, both the humanized antibody and early stage vaccination studies suggest that use of leukotoxins as immunizing agents is a potentially reasonable approach toward promoting natural clearance of infection. Blocking leukotoxin activity will necessitate targeting multiple toxins, as not all strains contain the same leukotoxin profile and each toxin appears to contribute variably to different disease states. A better understanding of leukotoxin mode(s) of action in vivo, inherent redundancies or lack thereof, and the intricacies of cellular targeting will prove beneficial in our ability to initiate the development of novel strategies to counter *S. aureus* infection.
